# Fgfr3 Is a Transcriptional Target of Ap2δ and Ash2l-Containing Histone Methyltransferase Complexes

**DOI:** 10.1371/journal.pone.0008535

**Published:** 2009-12-31

**Authors:** Cheryl C. Tan, Martin J. Walsh, Bruce D. Gelb

**Affiliations:** 1 Department of Biological Chemistry, University of California Los Angeles School of Medicine, Los Angeles, California, United States of America; 2 Department of Pediatrics, Mount Sinai School of Medicine, New York, New York, United States of America; 3 Department of Genetics and Genomic Sciences, Mount Sinai School of Medicine, New York, New York, United States of America; 4 Department of Structural and Chemical Biology, Mount Sinai School of Medicine, New York, New York, United States of America; 5 Center for Molecular Cardiology, Mount Sinai School of Medicine, New York, New York, United States of America; Ludwig-Maximilians-Universität München, Germany

## Abstract

Polycomb (PcG) and trithorax (trxG) proteins play important roles in establishing lineage-specific genetic programs through induction of chromatin modifications that lead to gene silencing or activation. Previously, we described an association between the MLL/SET1 complexes and a highly restricted, gene-specific DNA-binding protein Ap2δ that is required for recruitment of the MLL/SET1 complex to target *Hoxc8* specifically. Here, we reduced levels of Ap2δ and Ash2l in the neuroblastoma cell line, Neuro2A, and analyzed their gene expression profiles using whole-genome mouse cDNA microarrays. This analysis yielded 42 genes that are potentially co-regulated by Ap2δ and Ash2l, and we have identified evolutionarily conserved Ap2-binding sites in 20 of them. To determine whether some of these were direct targets of the Ap2δ-Ash2l complex, we analyzed several promoters for the presence of Ap2δ and Ash2l by chromatin immunoprecipitation (ChIP). Among the targets we screened, we identified *Fgfr3* as a direct transcriptional target of the Ap2δ-Ash2l complex. Additionally, we found that Ap2δ is necessary for the recruitment of Ash2l-containing complexes to this promoter and that this recruitment leads to trimethylation of lysine 4 of histone H3 (H3K4me3). Thus, we have identified several candidate targets of complexes containing Ap2δ and Ash2l that can be used to further elucidate their roles during development and showed that *Fgfr3* is a novel direct target of these complexes.

## Introduction

PcG and trxG proteins act antagonistically to maintain heritable patterns of gene expression, with the former marking genes for repression and the latter for activation. PcG complexes are associated with trimethylation of histone H3 at lysine 27 (H3K27me3) whereas trxG complexes are linked to H3K4me3 [1], [2]. This relationship embodies the characteristic of cellular memory to establish the identity in each cell type during development. Previously, these marks were considered to be static; recent evidence, however, has shown that these marks are involved in dynamic gene regulation through active recruitment of PcG and trxG complexes during cellular differentiation [2], [3]. Studies using embryonic stem (ES) cells and neural and muscle progenitors reveal that these marks vary depending on the cell type and that the majority of these marks is present at the promoters of key developmental genes [3], [4]. Furthermore, experiments that are based on chromatin immunoprecipitation coupled to DNA microarray analysis (ChIP-chip) and the more recent ChIP-seq, in which enriched DNA is directly sequenced, reveal an association between the intensity of the H3K4me3 epigenetic mark at the promoter and active transcription [3]. Conversely, the presence of the H3K27me3 mark is associated with gene repression [3]. These data suggest that PcG and trxG proteins play a role in establishing lineage-specific genetic programs through induction of chromatin modifications.

The trxG protein group includes members of the MLL/SET1 family of histone lysine methyltransferases (HKMTs) and their associated proteins. The MLL/SET1 family consists of six members, Mixed Lineage Leukemia 1 (MLL1), MLL2 (ALR), MLL3 (HALR), MLL4, SET1A and SET1B, which share a catalytic SET domain that has been shown to have H3K4 methyltransferase activity [5], [6], [7], [8]. MLL/SET1 proteins exist in multimeric complexes that contain three highly conserved subunits: Ash2l, RbBP5 and WDR5 [9]. Recently, it had been reported that these subunits are important for regulating the enzymatic activity of the SET domain-containing factor. Ash2l, in particular, was shown to be critical for H3K4me3 as downregulation of Ash2l leads to a genome-wide decrease in this epigenetic mark [10].

We recently reported that the gene-specific transcription factor Activating protein 2δ (Ap2δ) is important for the recruitment of MLL2 to the *Hoxc8 locus during embryogenesis and that this recruitment leads to H3K4me3 and subsequent gene activation [11]. Ap2δ is a member of the Ap2 family of sequence-specific DNA-binding proteins, which consists of Ap2α, -β, -γ, -δ and -ε. Ap2 proteins bind a GC-rich consensus sequence that is found on a variety of cellular and viral enhancers. Ap2δ is considered the most divergent family member, as it has a unique transactivation domain (TAD) that has been shown to specifically bind Ash2l. Additionally, Ap2δ's gene, Tcfap2d, has a highly restricted expression pattern and is found in the developing myocardium, central nervous system and retina [12].*

To search systematically for Ap2δ- and Ash2l-regulated targets, we assessed the transcriptome using cDNA microarrays to identify genes whose expression was significantly decreased when either protein was diminished and then filtered those results using the criterion of an evolutionarily conserved Ap2-binding site in the promoter. To identify true targets of Ap2δ and Ash2l-containing complexes among several candidates, we tested for the presence of Ap2δ and Ash2l at their promoters. Among the genes we tested, we identified *Fgfr3* as a direct target of Ap2δ and Ash2l given that both proteins were present at the *Fgfr3* promoter and that downregulation of either Ap2δ or Ash2l resulted in a decrease of *Fgfr3* expression. Thus, we provide evidence suggesting that Ap2δ plays an important role in altering the epigenetic landscape of a set of developmentally regulated targets through recruitment of Ash2l-containing HKMT complexes.

## Results

### Identification of Ap2δ and Ash2l Target Genes by cDNA Microarray Analysis

**Figure 1:**
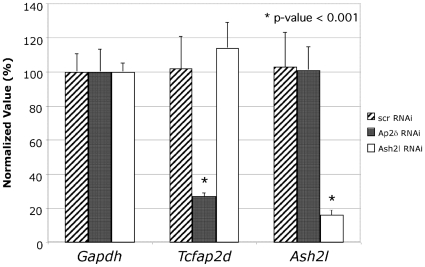
*Tcfap2d* and *Ash2l* are downregulated in Neuro2a cells treated with either Ap2δ or Ash2l RNAi. Total RNA was extracted 72 hours post-transfection from Neuro2a cells treated with *Tcfap2d*- or *Ash2l*-specific siRNA or scrambled control. *Gapdh*, *Tcfap2d* and *Ash2l* transcript levels were quantified by real-time PCR. Normalized values were calculated as percentages of transcript levels detected in cells treated with the scrambled control. Significant differences are as indicated with * (p≤.001).

To identify targets of Ap2δ and Ash2l, we performed whole genome analysis of cDNA expression levels obtained from Neuro2a cells treated with either *Tcfap2d-* or *Ash2l*-specific RNAi or a scrambled control. As shown in Fig. 1, treatment with either *Tcfap2d*- or *Ash2l*-specific RNAi resulted in a significant decrease of their respective transcripts only. Previously, we had shown that treatment with *Ash2l*-specific RNAi resulted in a decrease in Ash2l protein only [11].

**Figure 2:**
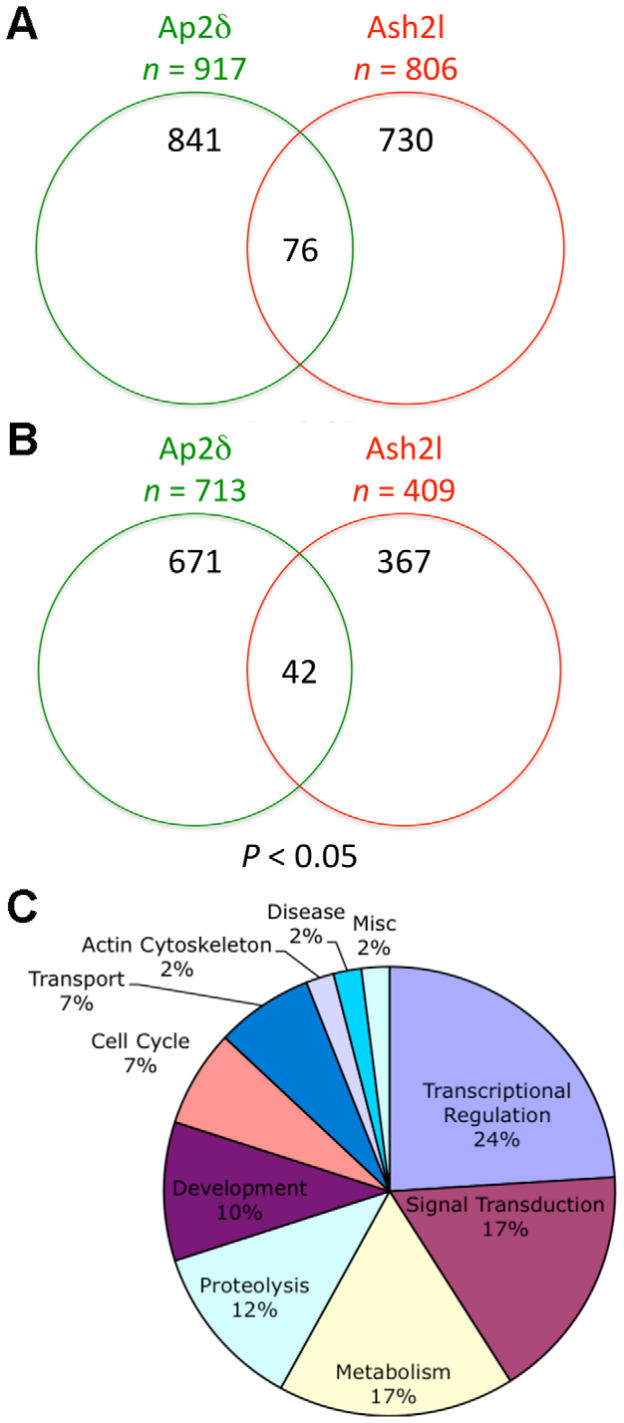
Ap2δ and Ash2l regulate a variety of genes involved in development. (*A*) Downregulation of *Tcfap2d* and *Ash2l* in cells leads to the differential expression of 917 and 806 genes, respectively. Comparison of these groups yields 76 genes whose expressions are changed when both *Tcfap2d* and *Ash2l* levels are reduced. (*B*) Among the 713 and 409 genes that are downregulated when *Tcfap2d* or *Ash2l* are decreased, respectively, 42 genes are decreased when both genes are reduced. (*C*) Functional annotation based on gene ontology (GO) reveal that the majority of the 42 genes encode for a variety of developmental proteins involved in transcriptional regulation and signal transduction. Microarray analysis was performed with cDNAs obtained from Neuro2a cells treated with either *Tcfap2d*- or *Ash2l*-specific RNAi or a scrambled control. Signal values were calculated using the MAS5 and PLIER statistical algorithms. Genes with a significance level of p<0.05 as compared to the scrambled control were selected for analysis.

To systematically identify genes regulated by both Ap2δ and Ash2l at the genome-wide scale, we obtained cDNAs from RNAi-treated Neuro2a cells and performed microarray analysis using the GeneChip® Mouse Genome 430 2.0 Array. Triplicate microarray experiments were performed comparing signals obtained from cells treated with either *Tcfap2d*- or *Ash2l*-specific RNAi to those of cells treated with a scrambled control. Signal values were calculated using the MAS5 and PLIER statistical algorithms. Genes that had a significance level of p<0.05 and a fold change greater than 1.1 were selected for analysis. Using signal values obtained from the MAS5 probe summarization algorithm, we identified 917 and 806 genes that were differentially expressed when Ap2δ or Ash2l, respectively, was downregulated. Comparison of these two groups yielded 76 genes whose expression was significantly altered when *Tcfap2d* and *Ash2l* were knocked down individually (Fig. 2*A*). Given that Ap2δ and Ash2l form a complex that is involved in H3K4me3, we assumed that a reduction in either Ap2δ or Ash2l would lead to decreased expression of their direct targets. Hence, to identify candidate targets of the Ap2δ-Ash2l complex, we focused solely on genes that were downregulated when they were reduced. Of the 76 genes whose expressed was significantly changed when Ap2δ and Ash2l were decreased, 33 genes with known function were downregulated. To determine whether we could identify additional targets that had not been previously identified by MAS5, we applied an alternative method using the PLIER probe summarization algorithm to obtain signal values. Through this method, we identified 9 additional genes that were downregulated when Ap2δ and Ash2l levels were decreased. Altogether, 42 genes were identified as candidate targets of the Ap2δ-Ash2l complex (Fig. 2*B*).

Functional annotation was performed based on gene ontology (GO) for the 42 genes that were downregulated in RNAi-treated cells (Table 1). A majority of the genes encodes for proteins that are involved in particular developmental functions, such as transcriptional regulation and signal transduction (Fig. 2*C*). Indeed, a significant enrichment of transcriptional regulators was identified in our analysis as only a small percentage (∼4%) of genes encode for this class of genes in the mouse genome [14]. Additionally, these gene functions are consistent with the role of both Ap2δ and Ash2l in development. We therefore concluded that these genes were probable targets of the Ap2δ-Ash2l complex. Altogether, these candidate targets may shed some insight into the role of the Ap2δ-Ash2l complex during development.

**Table 1:** Ap2δ and Ash2l Candidate Target Genes in Neuro2a Cells.

Gene Title	Gene Symbol	Accession Number	Number of motifs
***Transcription factors and regulators***			rVista*
expressed sequence AA407331	AA407331/Smad2	NM_010754.4	7
DAZ interacting protein 1-like	Dzip1l	NM_028258	
EBNA1 binding protein 2	Ebna1 bp2	NM_026932	1
eukaryotic translation initiation factor 2C, 3	Eif2c3	NM_153402	1
Kruppel-like factor 5	Klf5	NM_009769	3
leucine rich repeat and fibronectin type III domain containing 1	Lrfn1	NM_030562	8
nuclear factor I/X	Nfix	NM_001081981	12
zinc finger homeobox 3	Zfhx3	NM_007496	17
zinc finger protein 655	Zfp655	NM_028298	
zinc finger, MYM-type 6	Zmym6	NM_177462	
***Secreted factors***			
dickkopf homolog 3	Dkk3	NM_015814	
***Signal transduction***			
ATP-binding cassette, sub-family D (ALD), member 1	Abcd1	NM_007435	3
rho/rac guanine nucleotide exchange factor (GEF) 18	Arhgef18	NM_133962	
DIX domain containing 1	Dixdc1	NM_178118	4
fibroblast growth factor receptor 3	Fgfr3	NM_008010	8
gamma-aminobutyric acid (GABA-A) receptor, pi	Gabrp	NM_146017	
GTPase activating protein and VPS9 domains 1	Gapvd1	NM_025709	2
***Differentiation/Development/Tissue specific expression***			
crystallin, zeta	Cryz	NM_009968	
filamin, beta	Flnb	NM_134080	
low density lipoprotein receptor-related protein 4	Lrp4	NM_172668	6
plexin A3	Plxna3	NM_008883	6
***Nucleotide metabolism***			
arginine/serine-rich coiled-coil 2	Rsrc2	NM_001005525	2
***Protein metabolism***			
argininosuccinate lyase	Asl	NM_133768	
carbonic anhydrase 11	Car11	NM_009800	
Esterase D/formylglutathione hydrolase	Esd	NM_016903	
flavin containing monooxygenase 2	Fmo2	NM_018881	
histocompatibility (minor) HA-1	Hmha1	NM_027521	2
Zinc finger, DHHC domain containing 13	Zdhhc13	NM_028031	1
***Intracellular Transport***			
spire homolog 1 (Drosophila)	Spire1	NM_194355	
vacuolar protein sorting 13B (yeast)	Vps13b	AK122302	
***Ubiquitination***			
BRCA1/BRCA2-containing complex, subunit 3	Brcc3	NM_145956	
ubiquitin-activating enzyme E1, Chr Y 1	Ube1y1	NM_011667	
***Proteolysis***			
AE binding protein 1	Aebp1	NM_009636	
methionine aminopeptidase-like 1	Metapl1	NM_025633	1
protease, serine, 36	Prss36	NM_001081374	
***Actin Cytoskeleton Organization***			
coronin, actin binding protein, 2B	Coro2b	NM_175484	
***Transport***			
ATPase, class II, type 9A	Atp9a	NM_015731	8
***Cell Cycle/Apoptosis***			
cell division cycle and apoptosis regulator 1	Ccar1	NM_026201	
dendrin	Ddn	AK158894	4
Nuclear mitotic apparatus protein 1	Numa1	NM_133947	
***Disease***			
carnitine deficiency-associated gene expressed in ventricle 3	Cdv3	NM_175565	4
***Misc***			
follicular lymphoma variant translocation 1	Fvt1	NM_027534	

* Number of evolutionarily conserved Ap2-binding sites within 5 kb upstream and 2 kb downstream of the transcriptional start site (TSS).

### Prediction of Evolutionarily Conserved Ap2-Binding Sites in the Promoter Region of Putative Targets

To identify direct targets of the Ap2δ-Ash2l complex, we searched the promoters of the 42 candidate genes for evolutionarily conserved Ap2-binding sites using rVista 2.0 [15]. We analyzed genomic sequences up to 5 kilobases (kb) upstream and 2 kb downstream of the transcriptional start site (TSS). Of the 42 candidate targets tested, we found highly conserved Ap2-binding sites in 21 of 42 genes we assessed (Table 1). Given that a number of the candidate genes had Ap2-binding sites within 5 kb of the TSS, we concluded that these genes with Ap2-binding sites might be direct targets of the Ap2δ-Ash2l complex.

### Knockdown of Tcfap2d and Ash2l Leads to Decreased Expression of Candidate Ap2δ and Ash2l Targets

**Figure 3:**
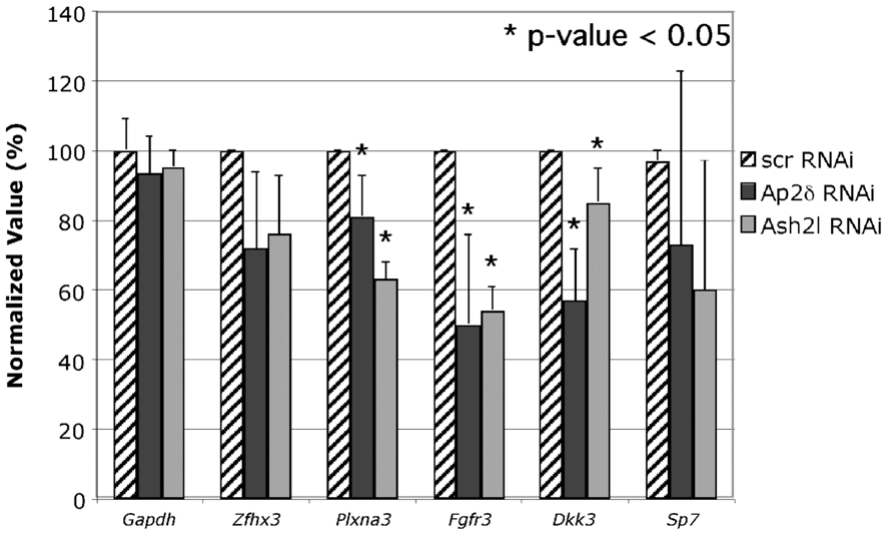
Ap2δ and Ash2l regulate *Plxna3, Fgfr3 and Dkk3* expression in Neuro2a cells. ***Plxna3***, ***Fgfr3*** **and** ***Dkk3*** **are downregulated when Ap2δ and Ash2l are downregulated.** Total RNA was extracted 72 hours post-transfection from Neuro2a cells treated with *Tcfap2d*- or *Ash2l*-specific siRNA or scrambled control. *Gapdh*, *Zfhx3, Plxna3, Fgfr3, Dkk3* and *Sp7* transcript levels were quantified by real-time PCR. Normalized values were calculated as percentages of transcript levels detected in cells treated with the scrambled control. Significant differences are as indicated with * (p≤.05).

To determine whether candidate targets with evolutionarily conserved Ap2-binding sites were indeed regulated by Ap2δ and Ash2l, we investigated the expression level of these targets upon downregulation of Ap2δ or Ash2l. We selected a number of genes that had roles in transcriptional regulation, development and signal transduction and tested the expression level of these genes using quantitative RT-PCR. Steady-state transcript levels for Plexin A3 (*Plxna3*), Fibroblast growth factor receptor 3 (*Fgfr3*) and Dickkopf homolog 3 (*Dkk3*) were significantly downregulated in Neuro2a cells after treatment with either *Tcfap2d*- or *Ash2l*-specific RNAi (Fig. 3). *Plxna3, Fgfr3* and *Dkk3* encode proteins that play important roles in neuronal development [16], [17], [18]. Ap2δ, in turn, has been implicated in neuronal development due to its highly restricted expression pattern in this tissue during embryogenesis [12]. Furthermore, MLL complexes have been implicated in neuronal differentiation, as MLL recruitment leads to increased H3K4me3 and activation of neuronal-specific genes [19]. Given that the candidate genes have overlapping roles in neuronal development with Ap2δ and Ash2l-containing complexes, we predicted that these candidate genes were likely to be direct targets of Ap2δ and Ash2l.

### Ap2δ Recruits Ash2l to the Fgfr3 Locus and Promotes H3K4me3

**Figure 4:**
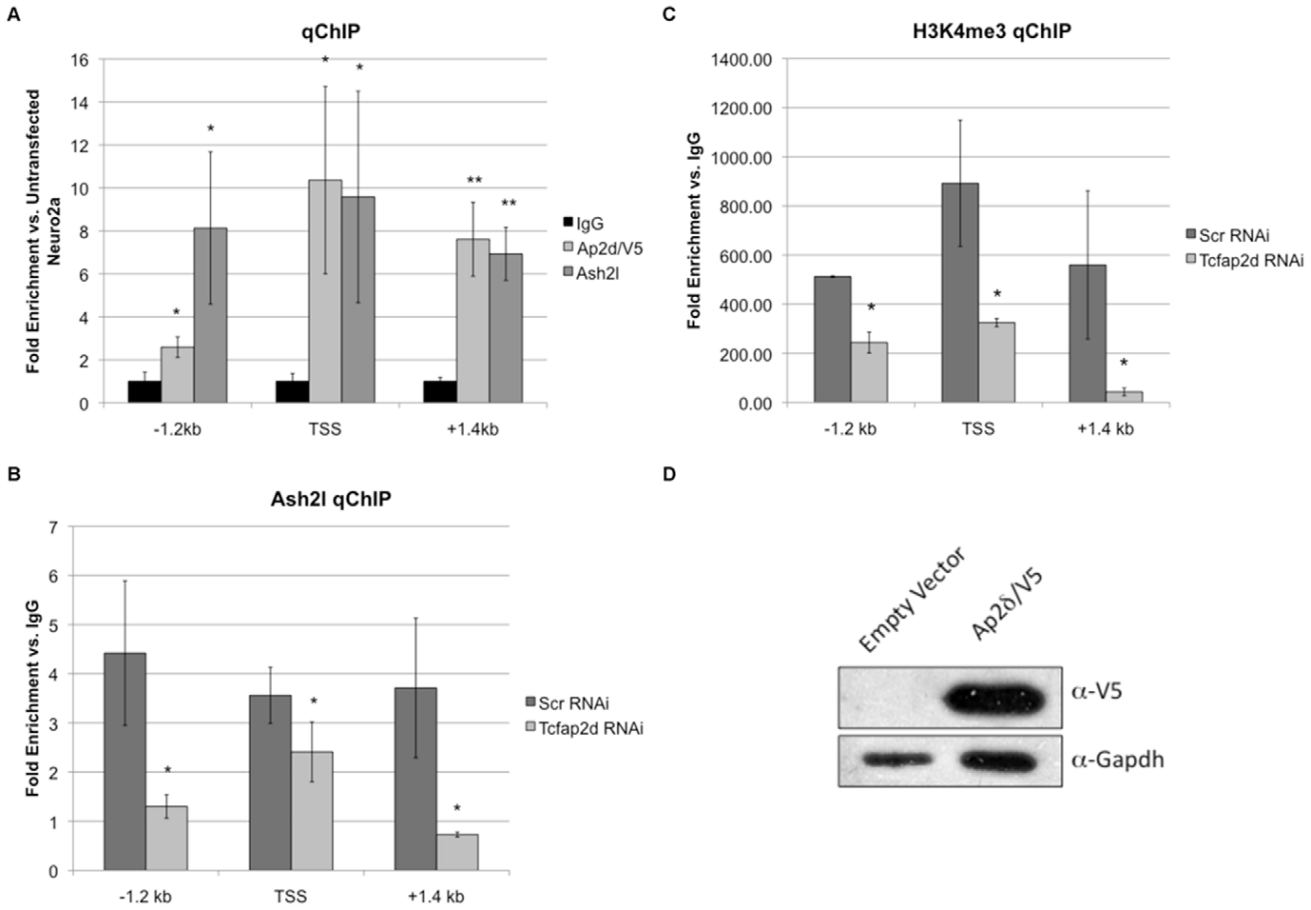
Ap2δ recruits Ash2l-containing HMT complexes to the *Fgfr3* locus in Neuro2a cells. (*A*) (*Top*) Ap2δ and Ash2l bind specific regions of the Fgfr3 promoter that are highly enriched in evolutionarily conserved Ap2-binding sites. The sites are located at the transcriptional start site (TSS) and regions ∼1.2 kb (−1.2 kb) upstream and ∼1.4 kb (+1.4 kb) downstream of the TSS. (*Bottom*) Western blot analysis show expression of Ap2δ/V5 in Neuro2a cells transfected with either an empty vector or an Ap2d/V5 expression construct. *(B*) Ap2δ downregulation results in decreased localization of Ash2l at the promoter concomitant with reduced HK4me3 (*C*). *(D)* Real-time PCR analysis shows a specific downregulation of *Tcfap2d* transcripts in Neuro2a cells treated with *Tcfap2d*-specific siRNA only. Normalized values were calculated as percentages of transcript levels detected in cells treated with the scrambled control. Significant differences are as indicated with * (p≤.05) and ** (p≤.005).

To identify direct targets of Ap2δ and Ash2l, we determined whether these proteins were present on the *Fgfr3, Plxna3* and *Dkk3* promoters. We hypothesized that Ap2δ and Ash2l would bind the promoters of these genes through highly conserved Ap2-binding sites that were previously identified *in silico*. To test this hypothesis, we performed chromatin immunoprecipitation (ChIP) using antibodies against V5/Ap2δ and Ash2l and analyzed the bound DNA by quantitative PCR. We found that Ap2δ and Ash2l were present only at the *Fgfr3* promoter. Additionally, these proteins co-localized at various regions of the promoter that were highly enriched in evolutionarily conserved Ap2-binding sites (Fig. 4*A*). These regions include the sites ∼1.2 kb (−1.2 kb) upstream and ∼200 bp (TSS) and ∼1.4 kb (+1.4 kb) downstream of the TSS.

To determine whether Ap2δ recruits Ash2l-containing histone methyltransferases to the *Fgfr3* promoter, we performed ChIP analysis with anti-Ash2l antibodies and Neuro2a cells treated with *Tcfap2d*-specific siRNA. Downregulation of Ap2δ significantly decreased the association of Ash2l with the −1.2 kb, TSS and +1.6 kb sites (Fig. 4*B*). Having demonstrated an Ap2δ-dependent recruitment of Ash2l, we next determined whether this recruitment altered H3K4 trimethylation at the *Fgfr3* locus, as this epigenetic status marks transcriptional initiation [20], [21]. We performed ChIP experiments using anti-trimethylated H3K4 antibodies with chromatin fragments obtained from Neuro2a cells treated with either *Tcfap2d*-specific siRNA or a scrambled control. We found significantly reduced levels of H3K4 trimethylation at the *Fgfr3* locus when Ap2δ was downregulated (Fig. 4*C*). These results indicate that Ap2δ recruits Ash2l to the *Fgfr3* promoter resulting in H3K4me3 and increased gene activation. It should be noted that downregulation of Ap2δ did not alter Ash2l and Alr protein levels similar to previous results [11].

## Discussion

In this study, we identified a number of candidate targets of Ap2δ and Ash2l by comparing the gene expression profiles of Neuro2a cells treated with *Tcfap2d*- or *Ash2l*-specific RNAi. Functional classification of probable Ap2δ- and Ash2l-regulated targets revealed an overrepresentation of genes involved in transcriptional regulation, signal transduction and development. We also identified a number of genes that were involved in the Wnt/β-catenin pathway (*Dixdc1, Dkk3, Lrp4, AA407331/Smad2*) and the small GTPase-mediated signaling pathway (*Arhgef1, Gapvd1*) implying a probable role of Ap2δ and Ash2l-containing complexes in these developmental pathways. Moreover, a significant portion of these candidate genes (21 out of 42) contained evolutionarily conserved Ap2-binding sites. We demonstrated that one of the three candidate genes we assessed, *Fgfr3*, was indeed a direct target of Ap2δ and Ash2l given that both proteins co-localized at the promoter and that downregulation of Ap2δ or Ash2l resulted in decreased *Fgfr3* expression. Given that we had only assessed a limited window for Ap2-binding sites and only those sites for Ap2δ-Ash2l binding, it should be noted that the genes for which we did not find any Ap2 binding sites might have binding sites further up- and downstream of the regions that we had assessed. As such, genes that were downregulated but for which we failed to find an Ap2-binding site are not necessarily false positives or indirectly regulated.

A growing body of evidence has shown that Fgfr1, -2 and -3 play important roles in the proliferation and differentiation of neural stem cells (NSCs). Immunocytochemical studies with NSCs derived from E15 rat striatum showed that expression of these receptors is developmentally regulated and cell lineage-specific. During the first day of culture, 50% and 70% of the NSCs or early precursors expressed *Fgfr1* and -*2*, respectively, while a restricted population expressed *Fgfr3* [22]. After 10 days in culture, the number of cells expressing *Fgfr1* and -*2* was significantly decreased to 15% of the total cell number whereas those expressing *Fgfr3* comprised a significant portion of the population suggesting that *Fgfr3* is increased during the process of cellular differentiation [22]. These results indicate that Fgfr3 may play a critical role in terminal differentiation while Fgfr1 and -2 may be important for earlier events, such as cell specification. Additionally, these Fgfr-positive cells were probed with various cell-lineage markers to determine whether there was an enrichment of a specific Fgfr in a particular cell lineage. *Fgfr1* and -*2* were detected in early oligodendroglial precursors whereas *Fgfr3* was detected in early oligodendroglial precursors, oligodendrocytes and astrocytes [22]. These data suggest that Fgfr1–3 play specific roles in the differentiation of NSCs into neurons, oligodendrocytes and astroctyes. To demonstrate that Fgfr1, 2 and 3 have roles in neuronal differentiation, NSCs were treated with basic FGF (bFGF), which is a ligand for these receptors. As predicted, treatment with exogenous bFGF resulted in increased proliferation of NSCs and an increased number of oligodendrocytes after seven days in culture [22]. Recently, mice were generated carrying various combinations of *Fgfr* mutant alleles to establish the role of Fgfr *in vivo* [*23*]. As predicted, mice with mutations in two or three *Fgfr* genes demonstrated patterning defects and increased apoptosis in the CNS, supporting the notion that Fgfr's are important for cell survival and identity [23]. Altogether, these data imply that Fgfr1, -2 and -3 play important roles in the ability of NSCs to self-renew and differentiate into distinct neuronal cell types. Moreover, their expressions in the developing CNS overlap with that of *Tcfap2d* confirming the role of Ap2d in *Ffgr3* regulation during development [24]. Indeed, these roles are consistent with those of trxG proteins whose functions have been linked to cell differentiation and memory.

Although we identified several Ap2δ- and Ash2l-regulated genes, a vast majority of differentially expressed genes did not overlap when the gene expression profiles were compared between cells treated with *Tcfap2d*-specific RNAi and those treated with *Ash2l*-specific RNAi. Given that Ash2l and its associated proteins are expressed ubiquitously, we hypothesized that Ash2l achieved its specificity through interactions with developmentally regulated transcription factors, such as Ap2δ. This would imply that Ash2l would have functions independent of those attributed to Ap2δ. Indeed, we found that only 76 out of 806 differentially expressed genes in Ash2l RNAi-treated cells overlapped with those in Ap2δ RNAi-treated cells. Similarly, Ap2δ may also interact with other co-activators, such as histone acetyltransferases and lysine demethylases, to activate its downstream targets. Previously, it had been reported that Ap2 proteins interacted with Cited2 and CBP to activate their targets indicating that Ap2δ may also associate with these factors in activating its downstream targets. This hypothesis is further supported by the observation that interactions with Cited2 and CBP occur in regions of the Ap2 protein that are nearly identical among Ap2 family members including Ap2δ. As such, Ap2δ's interaction with Ash2l may occur independently or in addition to its interaction with Cited2 and CBP. These interactions would, in turn, result in a repertoire of genes that are regulated by Ap2δ independently of Ash2l. Our results are consistent with this hypothesis, as only 76 out of 917 differentially expressed genes in Ap2δ RNAi-treated cells overlapped with those in Ash2l RNAi-treated cells.

It had been suggested that Ash2l and its associated proteins, including the MLL/SET1 subunits, are global regulators of gene expression given their expression patterns and developmental functions. As such, deletion of Ash2l or any of its associated proteins may result in either embryonic lethality or a pleiotropic defect that could potentially mask a variety of distinct developmental phenotypes. To circumvent this issue, one could potentially analyze the role of Ash2l through analysis of its various regulators, such as Ap2δ. Our studies are, therefore, an initial step in elucidating the function of Ash2l *in vivo*, providing a library of genes and pathways that are potentially regulated by Ash2l when it interacts with Ap2δ. Additionally, our studies in Neuro2a cells using endogenous proteins may reflect to a limited extent conditions similar to that of neural progenitors *in vivo*. Given that Neuro2a cells have oncogenic properties, these targets will need to be validated *in vivo*.

In conclusion, we have identified a library of genes that are regulated by both Ap2δ and Ash2l. A significant portion of these candidate target genes contains evolutionarily conserved Ap2-binding sites implying that several of them are direct targets of the Ap2δ and Ash2l-containing HMT complexes. Among the targets we screened, we identified *Fgfr3* as a novel target of both Ap2d and Ash2l. Thus, we provide evidence that these candidate genes will be useful in elucidating the developmental roles of Ap2δ and Ash2l.

## Materials and Methods

### Cell Culture and Immunoblotting

Neuro2a cells (ATCC, Manassas, VA) were maintained in Dulbecco's Modified Eagle Medium (Invitrogen) supplemented with 10% (v/v) fetal bovine serum and antibiotics (Invitrogen) at 37 °C with 5% CO_2_. Confluent cells were transfected with constructs expressing Ap2δ/V5 using Lipofectamine 2000 (Invitrogen) at a DNA-to-transfection reagent ratio of 1∶3. Cells were harvested in PBS after 48 h and incubated in lysis buffer (50 mM Tris-HCl pH 8.0, 1% Triton X-100, 150 mM NaCl, 10 mM EDTA) supplemented with 1X Protease Inhibitors (Roche, Indianapolis, IN). Lysates were resolved by 10% SDS-PAGE and electrotransferred onto nitrocellulose membranes. Immunoblots were probed with anti-V5 (Invitrogen) and anti-Gapdh antibodies (Sigma Aldrich, St. Louis, MO) that were detected by chemiluminescence according to protocol (Amersham Biosciences, Piscataway, NJ).

### RNA Analysis

For siRNA knockdown experiments, Neuro2a cells were transfected with *Tcfap2d*- or *Ash2l*-specific siRNA or a scrambled control using Dharmafect 1 (Dharmacon, Lafayette, CO), and total RNA isolated 72 h post transfection. Total RNA was extracted using Trizol reagent according to the manufacturer's protocol (Invitrogen) and reverse transcribed using Superscript™ III reverse transcriptase and oligo-dT primers (Invitrogen). Transcript levels were determined by real-time PCR using *Gapdh* as an internal control.

### Microarray Analysis

Total RNA was extracted from Neuro2a cells transfected with either *Tcfap2d-* or *Ash2l-*specific siRNA using the RNeasy Kit (Qiagen, Valencia, CA). Total RNA was reverse transcribed using a T7-oligo d(T) primer (Affymetrix, Santa Clara, CA), and cDNA was used as template for *in vitro* transcription using biotin-modified ribonucleotides. Biotinylated cRNA targets were fragmented and hybridized to Affymetrix GeneChip Mouse Genome 430 2.0 Arrays. Arrays were subsequently washed, stained and scanned using an Affymetrix GeneChip®-related software. ArrayAssist (Stratagene) was used to determine statistical significance among probe sets that were differentially expressed between gene-specific siRNA- and NTC-treated samples. Probe sets that were changed at least 1.1-fold with a p-value≤0.05 were used for further studies. Triplicate arrays were used for each sample to obtain statistical significance.

### Chromatin Immunoprecipitation (ChIP)

ChIP assays were performed according to manufacturer's protocol (Millipore Inc., Billerica, MA) [13]. Briefly, Neuro2a cells were transfected with Ap2δ/V5 or treated with RNAi for 72 h. Confluent cells were cross-linked in a solution containing 1% formaldehyde for 10 min at room temperature, and the reaction was terminated by the addition of glycine to a final concentration of 0.1 M. Cells were washed twice in ice-cold PBS with protease inhibitors (1 mM PMSF, 1 mg/ml aprotinin, 1 mg/ml pepstatin A) and harvested. Cells were lysed in SDS Lysis Buffer (Millipore) containing protease inhibitors for 10 min on ice. Lysates were sonicated to shear DNA into approximately 1-kb fragments. DNA-containing fractions were diluted 10-fold with a ChIP dilution buffer (Millipore) containing protease inhibitors. An equivalent amount of chromatin was incubated with anti-V5, -Ash2l, -trimethylated H3K4 (Millipore) or IgG antibodies overnight at 4°C. Immunoprecipitated material was collected with protein A agarose beads (Millipore) and washed sequentially with a low salt-immune complex wash buffer (Millipore), a high salt-immune complex wash buffer (Millipore), LiCl Immune Complex Wash Buffer (Millipore) and 1X TE (10 mM Tris-HCl, pH 8.0, 1 mM EDTA) for 5 min on a rotator at 4°C. Complexes were eluted from the Protein A-bound antibodies by addition of elution buffer (1% SDS, 0.1 M NaHCO_3_). Cross-linking reactions were reversed by heating at 65°C for 4 h. The DNA was recovered from immunoprecipitated material by proteinase K treatment at 55°C for 1 h followed by phenol/chloroform (1∶1) extraction, ethanol precipitation, and resuspension into 50 µl of nuclease-free water. Recovered DNA was analyzed by end-point and real-time PCR using the following loci-specific primers: Fgfr3 F1 5′-CCCTGGGGTGGCATCCTG-3′, Fgfr3 R1 5′-AAGGACCCCTCCCTGCAGACT-3′, Fgfr3 F2 5′-GACAGAGGAGACCCTGGAAAAGC-3′, Fgfr3 R2 5′- ATATCTCACCCCCTGACTGCTTCTG-3′, Fgfr3 F3 5′-GAGATGAGGGGCGGTTGTCC-3′ and Fgfr3 R3 5′-GTGGCTCCCTTTCGCATCCTT-3′.
